# The triglyceride-glucose index is associated with a higher risk of hypertension: evidence from a cross-sectional study of Chinese adults and meta-analysis of epidemiology studies

**DOI:** 10.3389/fendo.2025.1516328

**Published:** 2025-02-24

**Authors:** Xiaoli Ren, Mengying Chen, Liyou Lian, Huimin Xia, Wei Chen, Shengjie Ge, Lijuan Yang, Qingxi Jiang, Xuejiang Gu, Bo Yang, Xiang Hu

**Affiliations:** ^1^ Department of Endocrine and Metabolic Diseases, the First Affiliated Hospital of Wenzhou Medical University, Wenzhou, China; ^2^ The Laboratory Animal Center, Wenzhou Medical University, Wenzhou, China; ^3^ The First School of Medicine, School of Information and Engineering, Wenzhou Medical University, Wenzhou, China; ^4^ MAFLD Research Center, Department of Hepatology, The First Affiliated Hospital of Wenzhou Medical University, Wenzhou, China; ^5^ Department of Preventive Medicine, School of Public Health and Management, Wenzhou Medical University, Wenzhou, China; ^6^ Institute of Lipids Medicine, Wenzhou Medical University, Wenzhou, China

**Keywords:** hypertension, triglyceride-glucose index, insulin resistance, meta-analysis, cross-sectional analysis

## Abstract

**Background:**

The results of population-based studies show a diverse association between the triglyceride-glucose (TyG) index and hypertension. The present study aimed to investigate this association based on a cross-sectional study on Chinese adults and meta-analysis of epidemiology studies.

**Methods:**

The cross-sectional analysis used the baseline data from the on-going REACTION study in China. The TyG index was calculated as Ln [triglyceride(mg/dl)×fasting plasma glucose(mg/dl)/2]. A multivariate-adjusted logistic regression model was used to calculate the odds ratio (OR) with a 95% confidence interval (CIs) for the prevalence of hypertension, with the lowest TyG quartile as a reference.

**Results:**

A total of 4,177 participants aged 58.62 ± 8.40 years were included. TyG was significantly associated with higher odds of hypertension (OR:1.273, 95% CI:1.171-1.384), and the association remained pronounced with isolated systolic hypertension (OR:1.161, 95% CI:1.045-1.289) and systolic-diastolic hypertension (OR:1.313, 95% CI:1.163-1.483) but not with isolated diastolic hypertension (OR:1.164, 95% CI:0.929-1.460). In the present meta-analysis, 34 relevant studies were included through systematic searches of PUBMED, Embase, and the Cochrane Library. A positive association between the TyG index and hypertension was revealed in the meta-analysis of cohort studies (HR:1.57, 95% CI:1.25-1.96) and cross-sectional studies (OR:2.01, 95% CI:1.47-2.76).

**Conclusion:**

Elevated TyG index levels were significantly associated with a higher risk of clinical hypertension, which may provide new insights into the clinical management of hypertension.

## 1 Introduction

Elevated blood pressure (BP) is a leading risk factor for cardiovascular death (1, 2). Data from a Chinese national survey of 1.7 million adults showed that more than 40% of Chinese adults may have suffered from hypertension and the risk of high BP increases with age (3). In consideration of the improvement of awareness, treatment, and control of hypertension in middle-aged and elderly Chinese (4), identification of a new onset of hypertension may be effective in reducing the healthcare burden from cardiometabolic diseases in China.

Disorders of glucose and lipid metabolism, particularly elevated levels of triglyceride (TG) and fasting plasma glucose (FPG), are known risk factors for hypertension (5, 6). Mechanistic evidence to date has indicated that insulin resistance (IR) is linked with hypertension (7). Elevated levels of TG and FPG separately reflect IR status in the liver and adipose cells (8), with a general acceptance that conditions involved in high BP include endothelial dysfunction (9), pro-inflammatory response (10), and over-activation of the renin-angiotensin system (RAS) (11). The triglyceride-glucose (TyG) index, a composite parameter based on TG and FPG levels, presents a novel and cost-effective tool for assessing insulin resistance. TyG not only reflects glucose metabolism, but also captures lipid metabolism aspects that were not covered by traditional indices such as HOMA-IR. Its convenience due to only requiring routine biochemical parameters (12, 13) means it can be implemented more easily in clinical cardiology practice and resource-limited areas (14).

The TyG index may have the potential to become a novel indicator in detecting new-onset myocardial infarction (8) and metabolic syndrome (15). Nevertheless, evidence to date suggests that findings on using the TyG index to predict incident hypertension were inconsistent in several prospective cohorts (16, 17). Of note, two meta-analyses found the TyG index to be related to an increased risk of high BP (18, 19). One possibility was that the eligible selections from most cross-sectional studies may decrease the inference of causal association in pooled estimations and the inclusion of articles limited to Chinese regions may reduce population representativeness and generalizability. Moreover, the disagreements reported in previous studies were likely to be partially influenced by patients with different subtypes of hypertension including isolated systolic hypertension (ISH), isolated diastolic hypertension (IDH), and systolic-diastolic hypertension (SDH), low statistical powers attributed to small sample size, and insufficient adjustments for covariates.

To present population-based evidence with a large sample size and multiple covariates adjusted for, we used the baseline data from the REACTION study (20) to examine a cross-sectional association between the TyG index and hypertension in middle-aged and elderly Chinese patients. Moreover, a meta-analysis of data from prospective cohorts was further conducted to strengthen the observation at the causal evidence level.

## 2 Methods

### 2.1 Cross-sectional study design

#### 2.1.1 Study population

The present study was one part of the baseline survey from the REACTION study that aimed to investigate the association between diabetes and cancer among 259,657 adults aged over 40 years old in 25 local communities across different regions of China from 2011 to 2012 (20). Participants in the current study were selected from four communities in Wenzhou, Zhejiang Province. Eligible study participants were identified from local residence registration records and were required to be 40 years or older, with no restrictions on sex or ethnicity. Exclusion criteria for the participants were as follows: (1) severe hepatic or renal dysfunction (viral hepatitis, liver cirrhosis, autoimmune liver disease, chronic nephritis, and nephrotic syndrome), severe cardiovascular or cerebrovascular disease (myocardial infarction, coronary heart disease, and stroke), tumor, and psychiatric disorder; (2) data on key variables were missing. A total of 4,177 participants were included in the final analysis.

This study was conducted in accordance with the principles of the Declaration of Helsinki and approved by the Ruijin Hospital Ethics Committee, Shanghai Jiaotong University School of Medicine (201114RHEC). All participants signed informed consent forms prior to the study.

#### 2.1.2 Data collection

All participants completed standardized questionnaires, anthropometric tests, and laboratory examinations, which were conducted by professional physicians. The questionnaires included demographic characteristics, socioeconomic status, lifestyle factors (smoking, drinking, physical activity, and sedentary behavior), and medical history, and their specific definitions have been described in previous publications (20). Anthropometric tests were carried out by trained staff using uniform instruments (20, 21). Height and weight were measured with the participants wearing light clothing and no shoes. Body mass index (BMI) was calculated as weight (kg)/height (meters) squared. BP was measured using a mercury sphygmomanometer. Blood samples were collected from subjects who had fasted overnight for at least 8 hours. Routine biochemical data including FPG, 2-hour plasma glucose (2hPG), glycated hemoglobin A1c (HbA1c), total cholesterol (TC), TG, high-density lipoprotein cholesterol (HDL-C), and low-density lipoprotein cholesterol (LDL-C) were examined under a strict quality control (20, 21).

#### 2.1.3 Definitions

The TyG index was calculated according to the formula with Ln [TG (mg/dl)×FPG (mg/dl)/2] (22). Hypertension was defined as the objective measurement with systolic BP (SBP) ≥ 140mmHg and/or diastolic BP (DBP) ≥ 90mmHg (23) and/or the current use of antihypertensive medications. ISH was defined as SBP ≥ 140mmHg and DBP < 90mmHg (24). IDH was defined as SBP < 140mmHg and DBP ≥ 90mmHg (24). SDH was defined as SBP ≥ 140mmHg and DBP ≥ 90mmHg (24). A BMI of ≥ 24 kg/m² was defined as overweight/obesity according to the definition proposed by the Working Group on Obesity in China (25). Diabetes was diagnosed as FPG ≥ 7.0 mmol/L, 2hPG ≥ 11.1 mmol/L, or receiving hypoglycemic therapy (26). Dyslipidemia was defined as TC ≥ 5.18 mmol/L and/or TG ≥ 1.7 mmol/L and/or LDL-C ≥ 3.37 mmol/L and/or HDL-C < 1.04 mmol/L and/or on specific treatment for dyslipidemia (27). Depressive symptoms were measured by the Patient Health Questionnaire-9 (PHQ-9), and a PHQ-9 score ≥ 5 defined the presence of depressive symptoms (28).

#### 2.1.4 Statistical analysis

Data were presented as frequencies and percentages for categorical variables and mean ± standard deviation (SD) or median and interquartile range (IQR) for continuous variables according to their normality. Differences between groups were measured by the chi-square test or Fisher’s exact test for categorical variables and Student’s t-test or the Mann–Whitney U test for continuous variables. Participants were divided into four groups according to TyG index quartile levels, with the lowest quartile as a reference. A logistic regression model was used to calculate the odds ratio (OR) with a confidence interval (CI) for the prevalence of hypertension, with multiple adjustments for covariates including age, sex, education levels, lifestyle risk factors (smoking status, drinking status, physical activity, and sedentary behavior), and concomitant diseases (overweight/obesity, diabetes, dyslipidemia, and depressive symptoms). Subgroup analyses were conducted to determine if the TyG index interacted with age, sex, lifestyle risk factors, or concomitant diseases. Two sensitivity analyses were performed to test if the direction of the estimated association for the TyG index changed when (1) re-defining hypertension as SBP ≥ 130mmHg or DBP ≥ 80mmHg or (2) after additional adjustments for anti-hypertensive medications.

### 2.2 Meta-analysis design

In the additional meta-analyses, relevant population-based studies were included according to the following criteria: (1) population-based cross-sectional or cohort studies; (2) ORs or HRs with 95% CIs for the TyG index in relation to hypertension; (3) with systolic/diastolic BP greater than 140/90 mmHg or the use of antihypertensive medications as the diagnosis for hypertension.

The 9-star Newcastle–Ottawa Scale (NOS) (29) was used to evaluate the study quality of the included studies. Studies with an NOS score of 8 to 9 stars were considered high quality, 6 to 7 stars were moderate quality and 1 to 5 stars were low quality.

A random effects model was used to calculate the summary ORs for the cross-sectional studies and HRs for the prospective cohort studies. Heterogeneity was assessed using Q statistics and the I^2^ index (30). A sensitivity analysis was conducted to assess the robustness of the pooled association estimations by excluding one individual study at a time (31). Funnel plots, Egger’s test, and Begg’s test were used to evaluate the potential publication bias (32). All statistical analyses were performed using Stata version 17.0, with a *P-*value less than 0.05 indicating statistical significance.

## 3 Results

### 3.1 Characteristics of the individuals who participated in the cross-sectional study

Characteristics of the participants in the cross-sectional study are shown in **Table 1**. A total of 4,177 eligible participants (1,267 men and 2,910 women) were included, with a mean age of 58.62 ± 8.40 years old. The study population included 2,081 hypertensive patients and 2,096 individuals with normal blood pressure. Among the hypertensive patients, 924 were taking anti-hypertensive medication. The prevalence of hypertension was 49.82% (95% CI: 48.30-51.34), with a significant increase in the proportions of hypertension across the TyG quartiles (*P for trend* < 0.001). Participants in the highest TyG quartiles had a higher proportion of overweight/obesity, diabetes, and dyslipidemia than those in the lowest quartiles (all *P*<0.001). Moreover, compared to participants in the lowest TyG quartile, those in the higher TyG quartiles were more likely to be male, older, current smokers, and drinkers (all *P*<0.001).

**Table 1:** Descriptive characteristics of the 4,177 individuals who participated in the cross-sectional study.

Characteristic	TyG index				P for trend
	T1, n=1,044 (<8.44)	T2, n=1,045 (8.44-8.82)	T3, n=1,044 (8.82-9.24)	T4, n=1,044 (>9.24)	
Sociological factors					
Age (≥ 60, %)	341 (32.7)	472 (45.2)	523 (50.1)	555 (53.2)	<0.001
Sex (male, %)	219 (21.0)	305 (29.2)	316 (30.3)	427 (40.9)	<0.001
Education, %	553 (53.0)	552 (52.8)	516 (49.4)	540 (51.7)	0.302
Lifestyle risk factors					
Current smoking, %	92 (8.8)	113 (10.8)	136 (13.0)	222 (21.3)	<0.001
Current drinking, %	90 (8.6)	103 (9.9)	123 (11.8)	193 (18.5)	<0.001
Physical activity, %	245 (23.5)	252 (24.1)	247 (23.7)	201 (19.3)	0.024
Sedentary behavior, %	428 (41.0)	412 (39.4)	434 (41.6)	450 (43.1)	0.214
Concomitant disease					
Overweight/obesity, %	340 (32.6)	494 (47.3)	606 (58.0)	703 (67.3)	<0.001
Hypertension, %	344 (33.0)	477 (45.6)	583 (55.8)	677 (64.8)	<0.001
Diabetes, %	110 (10.5)	184 (17.6)	294 (28.2)	564 (54.0)	<0.001
Dyslipidemia, %	567 (54.3)	741 (70.9)	963 (92.2)	1036 (99.2)	<0.001
Depressive symptoms, %	50 (4.8)	59 (5.6)	63 (6.0)	46 (4.4)	0.805
Laboratory indicators					
FPG (mmol/L)	5.1 (4.8-5.5)	5.3 (5.0-5.8)	5.6 (5.2-6.2)	6.3 (5.5-7.9)	<0.001
2hPG (mmol/L)	6.3 (5.4-7.4)	6.9 (5.8-8.6)	7.6 (6.2-10.1)	9.8 (7.4-13.7)	<0.001
HbA1c (%)	5.6 (5.3-5.8)	5.7 (5.4-6.0)	5.9 (5.6-6.3)	6.3 (5.8-7.2)	<0.001
TC (mmol/L)	5.1 (4.5-5.7)	5.3 (4.7-6.0)	5.6 (4.9-6.2)	5.7 (5.0-6.5)	<0.001
TG (mmol/L)	0.8 (0.7-1.0)	1.3 (1.2-1.4)	1.8 (1.6-2.1)	2.9 (2.4-3.7)	<0.001
HDL-C (mmol/L)	1.6 (1.4-1.8)	1.4 (1.2-1.6)	1.3 (1.2-1.5)	1.2 (1.1-1.4)	<0.001
LDL-C (mmol/L)	2.9 (2.4-3.4)	3.3 (2.7-3.8)	3.3 (2.8-3.9)	3.1 (2.5-3.8)	<0.001

FPG, fasting plasma glucose; 2hPG, 2-hour plasma glucose; HbA1c, glycated hemoglobin A1; TC, total cholesterol; TG, triglyceride; HDL-C, high-density lipoprotein cholesterol; LDL-C, low-density lipoprotein cholesterol. Data are presented as median (interquartile range) or N (%). P-values were determined using the chi-square test for categorical variables and the Mann–Whitney U test for continuous variables.

### 3.2 Cross-sectional association between the TyG index and the prevalence of hypertension

There was a positive association between the TyG index and hypertension in all the statistical models with multiple adjustments (**Table 2**). Specifically, participants in the highest TyG quartile had higher odds of hypertension than those in the lowest TyG quartile (OR:1.273, 95% CI:1.171-1.384, *P*<0.001), and the associations were found to be pronounced in ISH (OR:1.161, 95% CI:1.045-1.289, *P*=0.005) and SDH (OR:1.313, 95% CI:1.163-1.483, *P*<0.001) but not in IDH (OR: 1.164, 95% CI:0.929-1.460, *P*=0.187).

**Table 2:** The association between the TyG index and the prevalence of hypertension in the cross-sectional study.

TyG index	Cases/participants		Model 1		Model 2		Model 3		Model 4	
			OR (95%CI)	P value	OR (95%CI)	P value	OR (95%CI)	P value	OR (95%CI)	P value
Hypertension	T1 (ref)	344/1,044								
	T2	477/1,045	1.709 (1.431-2.041)	<0.001	1.512 (1.257-1.820)	<0.001	1.513 (1.257-1.822)	<0.001	1.280 (1.054-1.555)	0.013
	T3	583/1,044	1.604 (1.468-1.753)	<0.001	1.499 (1.367-1.644)	<0.001	1.508 (1.375-1.655)	<0.001	1.289 (1.157-1.435)	<0.001
	T4	677/1,044	1.554 (1.463-1.651)	<0.001	1.465 (1.375-1.561)	<0.001	1.481 (1.389-1.579)	<0.001	1.273 (1.171-1.384)	<0.001
ISH	T1 (ref)	135/1,044								
	T2	226/1,045	1.858 (1.472-2.346)	<0.001	1.609 (1.263-2.051)	<0.001	1.596 (1.251-2.036)	<0.001	1.447 (1.124-1.862)	0.004
	T3	270/1,044	1.533 (1.368-1.717)	<0.001	1.409 (1.252-1.585)	<0.001	1.418 (1.259-1.596)	<0.001	1.279 (1.116-1.467)	<0.001
	T4	282/1,044	1.356 (1.257-1.462)	<0.001	1.264 (1.167-1.368)	<0.001	1.266 (1.169-1.371)	<0.001	1.161 (1.045-1.289)	0.005
IDH	T1 (ref)	25/1,044								
	T2	31/1,045	1.246 (0.731-2.126)	0.419	1.277 (0.743-2.196)	0.377	1.274 (0.739-2.194)	0.384	1.114 (0.637-1.947)	0.705
	T3	42/1,044	1.307 (1.017-1.681)	0.037	1.384 (1.071-1.789)	0.013	1.393 (1.078-1.801)	0.011	1.225 (0.917-1.636)	0.169
	T4	39/1,044	1.165 (0.983-1.381)	0.078	1.239 (1.036-1.483)	0.019	1.241 (1.036-1.487)	0.019	1.164 (0.929-1.460)	0.187
SDH	T1 (ref)	90/1,044								
	T2	122/1,045	1.401 (1.052-1.867)	0.021	1.316 (0.983-1.761)	0.065	1.318 (0.984-1.766)	0.064	1.121 (0.828-1.516)	0.459
	T3	166/1,044	1.416 (1.235-1.622)	<0.001	1.374 (1.196-1.578)	<0.001	1.377 (1.198-1.582)	<0.001	1.237 (1.054-1.452)	0.009
	T4	222/1,044	1.420 (1.301-1.550)	<0.001	1.374 (1.255-1.503)	<0.001	1.383 (1.263-1.515)	<0.001	1.313 (1.163-1.483)	<0.001

ISH, isolated systolic hypertension; IDH, isolated diastolic hypertension; SDH, systolic-diastolic hypertension; OR, odds ratio; 95% CI, 95% confidence interval. Hypertension was defined as SBP ≥ 140mmHg or DBP ≥ 90mmHg or on antihypertensive medication. ISH was defined as SBP ≥ 140mmHg and DBP < 90mmHg. IDH was defined as SBP < 140mmHg and DBP ≥ 90mmHg. SDH was defined as SBP ≥ 140mmHg and DBP ≥ 90mmHg. P-values were determined using the logistic regression. Model 1: Unadjusted. Model 2: Adjusted for age, sex and education levels. Model 3: Model 2 plus adjustment for lifestyle factors (current smoking, current drinking, physical activity, and sedentary behavior). Model 4: Model 3 plus adjustment for concomitant diseases (overweight/obesity, diabetes, dyslipidemia, and depressive symptoms).

**Figure 1:**
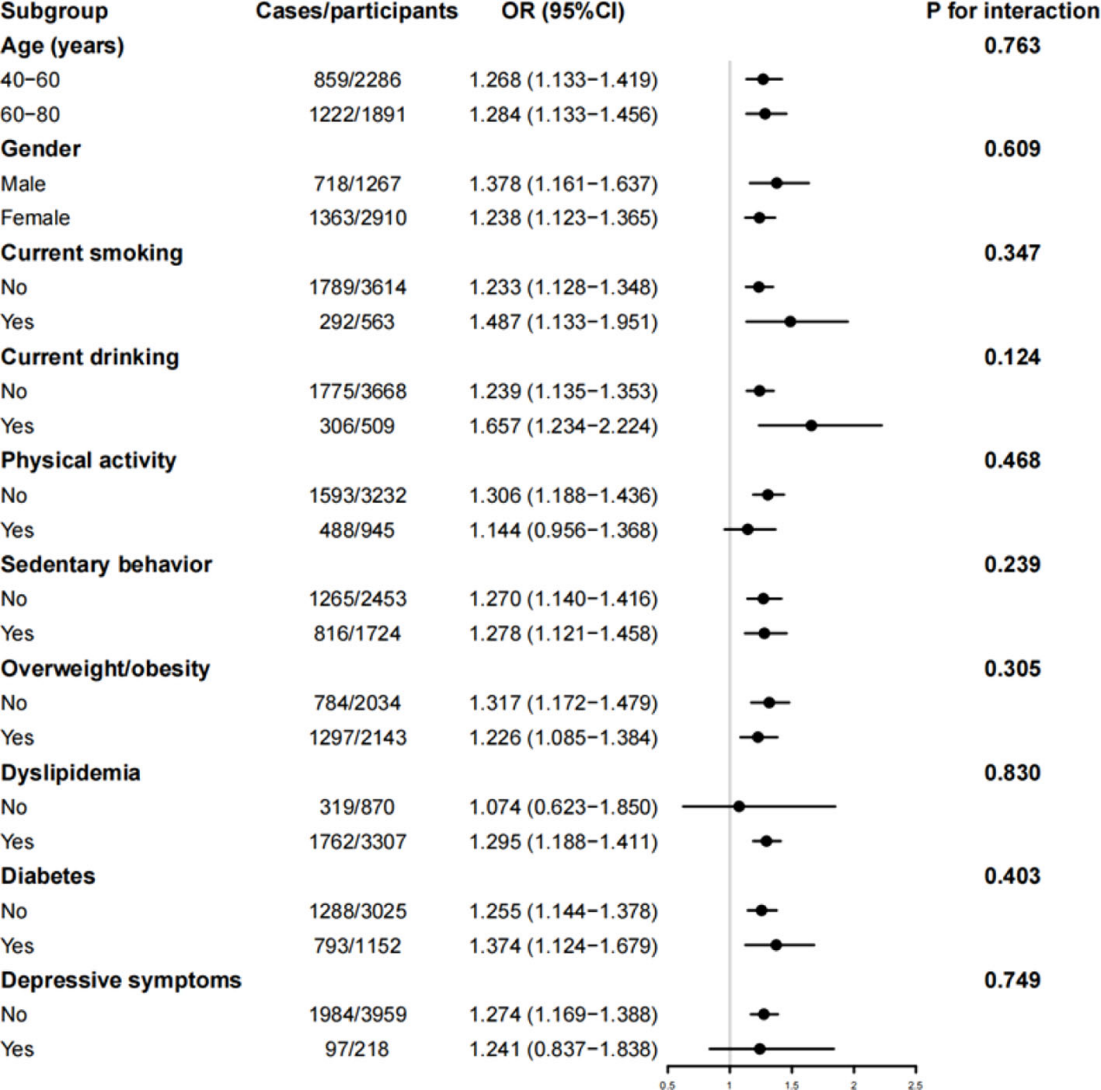
Subgroup analysis of the cross-sectional association between TyG index and hypertension. OR, odds ratio; 95% CI, 95% confidence interval. The model was adjusted for age, sex, education levels, current smoking, current drinking, physical activity, sedentary behavior, overweight/obesity, diabetes, dyslipidemia, and depressive symptoms.

In the subgroup analyses (**Figure 1**), the TyG index was found to have significant associations with the prevalence of hypertension in most of the subgroups, with no significant interactions between the TyG index and the strata for hypertension.

The results of the sensitivity analyses in which we defined hypertension as SBP ≥ 130mmHg and (or) DBP ≥ 80mmHg showed that the positive association between the TyG index and hypertension was not remarkably changed (**Supplementary Table S1**). Additionally, the results remained largely unchanged when anti-hypertensive medications were included in the model adjustment. Compared to those in the lowest TyG quartile, the participants in the highest TyG quartile had higher odds of hypertension (OR:1.249, 95% CI:1.130-1.381, *P*<0.001). This association was more pronounced in ISH (OR:1.136, 95% CI:1.019-1.266, *P*=0.021) and SDH (OR:1.275, 95% CI:1.126-1.444, *P*<0.001) but was not significant in IDH (OR: 1.136, 95% CI:0.898-1.437, *P*=0.289) (**Supplementary Table S1**).

### 3.3 Association between the TyG index and hypertension in the meta-analysis

The flowchart of the literature selection is shown in **Supplementary Figure S1**. One eligible study included two independent cohorts, thus 18 cohorts (16, 17, 33–47) and 18 cross-sectional studies (48–64) (17 cross-sectional studies and our current study) were eligible. The pooling analyses of the 18 cohorts included 210,694 participants with a median age of 49.1 years and a median follow-up period of 6.0 years, while the meta-analysis of the cross-sectional studies included 810,839 participants with a median age of 51.4 years. All 18 cohorts were of high quality except for seven articles of moderate quality (37, 39, 41, 43, 44, 46, 47). Among the 18 cross-sectional studies, 16 were of high quality and two were of moderate quality (50, 54).

**Figure 2:**
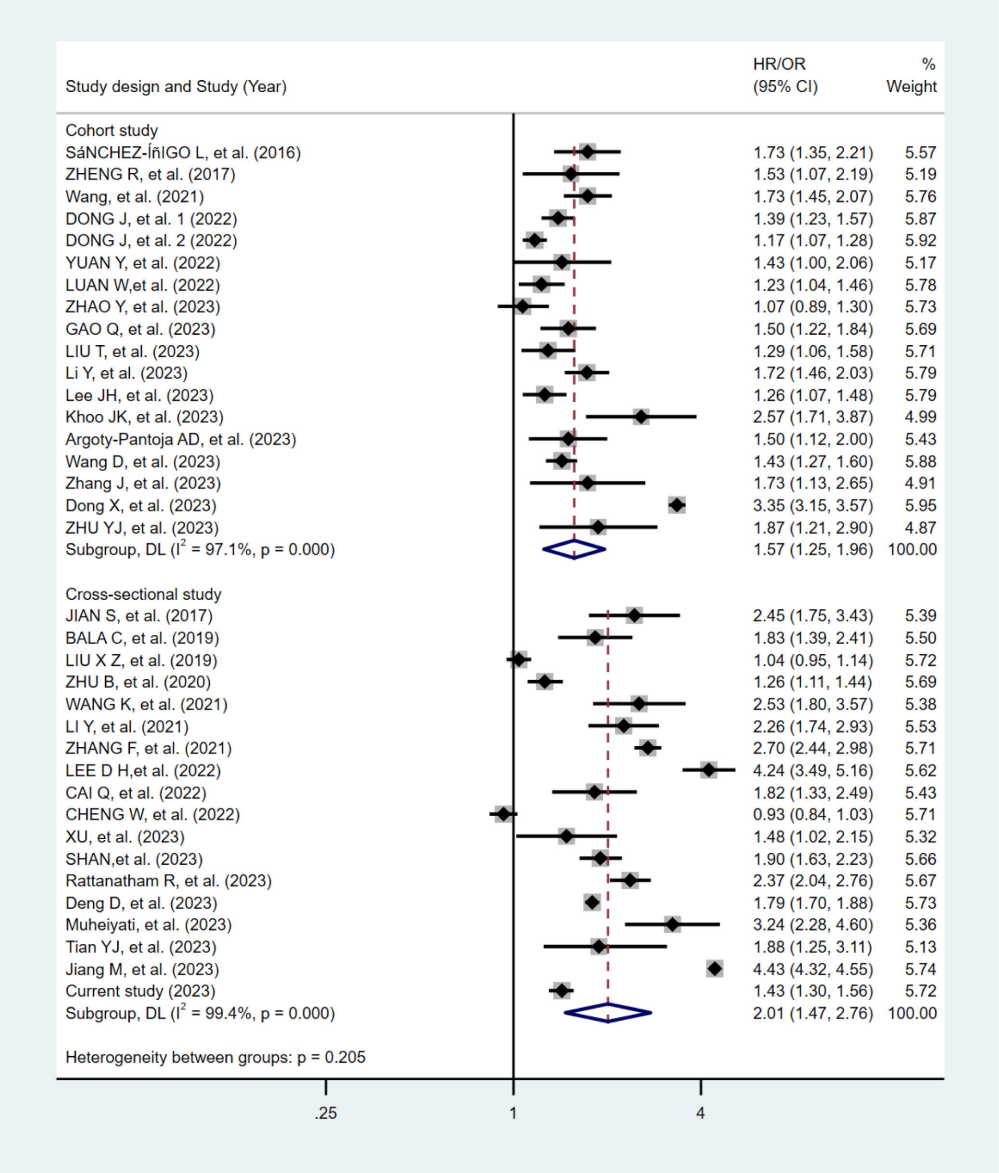
Forest plots of the association between the TyG index and hypertension in the meta-analysis.

The meta-analytic results from the random-effects models are shown in **Figure 2**. Participants with the highest TyG index had a higher risk of hypertension compared with those with the lowest, both in the meta-analysis of cohort studies (HR:1.57, 95% CI:1.25-1.96, I^2^ = 97.1%), and in the meta-analysis of cross-sectional studies (OR:2.01, 95% CI:1.47-2.76, I^2^ = 99.4%).

In the sensitivity analyses after excluding one article at a time, the pooled association estimates did not significantly change (**Supplementary Figures S2**, **S3**). In the subgroup analyses, no significant interactions were observed both in the meta-analysis of the cohort studies (**Supplementary Table S3**) and cross-sectional studies (**Supplementary Table S4**). The meta-analytic findings from cohort studies had no potential publication bias in the visual funnel plots (**Supplementary Figure S4**), Egger’s test (**Supplementary Figure S6**), or Begg’s test (**Supplementary Figure S8**). In contrast, potential publication bias was observed in these assessments for the meta-analytic findings from cross-sectional studies (**Supplementary Figures S5**, **S7**, **S9**).

## 4 Discussion

Our cross-sectional data demonstrated a significant positive association between the TyG index and hypertension in a middle-aged and elderly Chinese population, and this significant association was independent of age, sex, lifestyle factors, and concomitant diseases. The findings were also supported by current meta-analyses of prospective cohorts, which may strengthen the robustness of the inference of a causal association.

Our current findings were consistent with most of the previous population-based reports. A cross-sectional study involving 43,591 participants aged over 40 years old found that increases in the TyG index were more statistically associated with hypertension than blood lipids or glycemic parameters alone such as TC, TG, HDL-C, LDL-C, HbA1c, and 2hPG (51). This result was also seen in another cross-sectional study including 1,777 participants aged over 40 years old (48), and the TyG index was observed to be more associated with ISH, but not IDH. In support of these findings, this study demonstrated a positive association between the TyG index and hypertension, especially in ISH and SDH. In contrast, a recent study involving 21,670 Chinese adults aged 18-75 years old revealed a positive association with IDH only (55). The discrepancy between these observational studies may be explained by the age of the enrolled population. IDH is generally more prevalent among young adults (65), whereas ISH is prevalent in the middle-aged and elderly population (66). In our study of middle-aged and elderly Chinese patients, the number of IDH cases was limited, which may have led to the observation towards null. In additional analyses, the TyG index’s association with hypertension remained significant in most of the subgroups analyzed, suggesting that the observed association was independent of age, sex, lifestyle risk factors, and concomitant diseases. In addition, the results of sensitivity analyses in which we re-defined hypertension as SBP/DBP ≥ 130/80 mmHg, showed that the positive association between the TyG index and hypertension did not change, indicating the robustness of the TyG index in detecting high BP.

In the meta-analyses, available data from the observational studies were pooled and the directions for their summary association were consistent in both the cohort and cross-sectional studies, thereby reinforcing the observation obtained from the present cross-sectional analysis of Chinese adults. Moreover, the summary association estimates, as summarized in either the cohort or cross-sectional studies, had high heterogeneity at the statistical level but they remained significant in most of the subgroups and the results of a meta-regression analysis did not detect a significant interaction effect with age, sex, and obesity status on hypertension. Of note, when stratified by age, the TyG index was found to be pronounced with a high risk of hypertension in younger, middle-aged, and elderly persons. These summary findings from the pooling cohorts may not only further solidify the strength of epidemiological evidence at the causal-association level but also reinforce the consistency of this association across various age groups.

A series of mechanistic evidence to date likely to explain why hyperglycemia and dyslipidemia are potential risk factors that may trigger the development of high BP (5, 6). First, elevated levels of TG may partially lead to vascular endothelial dysfunction and oxidative stress, thereby leading to vascular remodeling signals that may be associated with elevated systolic and pulse pressure (67). Second, hypertriglyceridemia may cause hypercoagulability by promoting coagulation and inhibiting fibrinolysis, which increases blood viscosity and peripheral vascular resistance (68). Third, hyperglycemia may promote glucose and sodium reabsorption in the proximal convoluted tubules, thereby generating sodium retention and increased extracellular fluid (69). Finally, TyG, as a potential indicator for IR, may have in part prompted the development of hypertension through multiple biological mechanisms such as excessive activations of RAS and inflammatory response (10, 11).

Our study is the first to combine a population-based study with a meta-analysis and summarize relevant articles published worldwide, verifying the generalizability of the TyG index in predicting hypertension risk across different regions. However, there are several limitations in our study. First, our study of Chinese adults is a cross-sectional study, therefore causal inferences cannot be made. However, we performed a meta-analysis of cohort studies, in which the direction of the meta-analytic results was found to be consistent with the findings from our cross-sectional study. Second, our cross-sectional study was conducted in the middle-aged and elderly population only, thereby leading to a lack of representativeness of the whole population of Chinese adults. However, when stratified by age in the meta-analysis of the published cohort studies, the TyG index had a positive association with the risk of hypertension in younger, middle-aged, and older adults. Third, medical history was determined by participants’ self-report, which may have affected the results due to recall bias. Fourth, the association observed in the cross-sectional study may have been affected by other unknown confounders, therefore the present findings must be interpreted with caution. Fifth, the study population was restricted to individuals from Wenzhou. Blood pressure-related data were not collected during the 3^rd^-, 5^th^-, and 10th-year follow-ups in this region. In future follow-ups, we will strive to enhance data collection in this regard, aiming to provide more robust evidence for cohort studies. Finally, our current meta-analysis of prospective cohorts may partially or at least enhance the reliability of the observed findings, but there was high heterogeneity and potential publication bias in the pooling analysis of the published cross-sectional analyses. Therefore, the results of summarized evidence from prospective cohorts were warranted in the present study.

## 5 Conclusions

In conclusion, evidence from the current cross-sectional study and meta-analyses showed that participants with elevated TyG index levels had a high risk of hypertension, indicating that individuals with impaired glucose tolerance and/or lipid metabolism should be alert to incident hypertension, especially ISH and SDH. Such findings may provide strong support for the TyG index to be used as an independent indicator in screening hypertension risk in clinical cardiology practice.

## Data Availability

The data are held in a secure, confidential database, which can only be assessed by members of the REACTION group. The data that support the findings of this study are available from the corresponding author upon reasonable request. Requests to access the datasets should be directed to XH, huxiang@wmu.edu.cn.
