# Supplementary material for: The triglyceride-glucose index is associated with a higher risk of hypertension: evidence from a cross-sectional study of Chinese adults and meta-analysis of epidemiology studies
Source: Front Endocrinol (Lausanne). 2025 Feb 24;16:1516328. doi: 10.3389/fendo.2025.1516328 (PMC11891255; doi:10.3389/fendo.2025.1516328)
Supplement: Supplementary file 1 [file DataSheet1.docx]

***Supplementary Material***

# Literature searching strategies

Literature search strategy in Pubmed: 85 (publication till 2023-11-26):

("fasting triglyceride-glucose index"[Title/Abstract] OR "fasting triglycerides-glucose index"[Title/Abstract] OR "triglyceride and glucose (TyG) index"[Title/Abstract] OR "triglyceride and glucose index"[Title/Abstract] OR "triglyceride glucose (TyG) index"[Title/Abstract] OR "triglyceride glucose index (TyG index)"[Title/Abstract] OR "triglycerides glucose (TyG) index"[Title/Abstract] OR "triglycerides-glucose index"[Title/Abstract] OR "TyG index"[Title/Abstract] OR "triglyceride-glucose index"[Title/Abstract]) AND ("Hypertension"[MeSH Terms] OR "blood pressure high"[Title/Abstract] OR "blood pressures high"[Title/Abstract] OR "high blood pressure"[Title/Abstract] OR "high blood pressures"[Title/Abstract])

Literature search strategy in Embase: 483 (publication till 2023-11-26):

('hypertension'/exp OR 'acute hypertension' OR 'arterial hypertension' OR 'blood pressure, high' OR 'cardiovascular hypertension' OR 'controlled hypertension' OR 'endocrine hypertension' OR 'high blood pressure' OR 'high renin hypertension' OR 'htn (hypertension)' OR 'hypertensive disease' OR 'hypertensive effect' OR 'hypertensive response' OR 'neurogenic hypertension' OR 'preexistent hypertension' OR 'salt high blood pressure' OR 'salt hypertension' OR 'secondary hypertension' OR 'systemic hypertension') AND ('triglyceride-glucose index'/exp OR 'fasting glucose and triglyceride (tyg index)' OR 'fasting glucose and triglycerides (tyg index)' OR 'fasting plasma glucose and triglycerides (tyg index)' OR 'fasting plasma glucose and triglycerides index' OR 'fasting triglyceride-glucose index' OR 'fasting triglycerides-glucose (tyg) index' OR 'fasting triglycerides-glucose index' OR 'glucose and triglycerides (tyg index)' OR 'glucose and triglycerides index' OR 'glucose-triglyceride index (tyg)' OR 'glucose-triglycerides index' OR 'triglyceride and glucose (tyg) index' OR 'triglyceride and glucose index' OR 'triglyceride x glucose (tyg) index' OR 'triglyceride-glucose (tyg) index' OR 'triglyceride-glucose index (tyg index)' OR 'triglycerides x glucose (tyg index)' OR 'triglycerides-glucose (tyg) index' OR 'triglycerides-glucose index' OR 'triglycerides-glucose index (tyg index)' OR 'triglycerides-glucose index (tyg)' OR 'tyg index')

Literature search strategy in Cochrane Library: 3 (publication till 2023-11-26):

(("Blood Pressure, High"):ti,ab,kw OR ("Blood Pressures, High"):ti,ab,kw OR ("High Blood Pressure"):ti,ab,kw OR ("High Blood Pressures"):ti,ab,kw OR ("hypertension"):ti,ab,kw) AND (("fasting triglyceride-glucose index"):ti,ab,kw OR ("fasting triglycerides-glucose index"):ti,ab,kw OR ("triglyceride and glucose (TyG) index"):ti,ab,kw OR ("triglyceride and glucose index"):ti,ab,kw OR ("triglyceride glucose (TyG) index"):ti,ab,kw OR ("triglyceride glucose index (TyG index)"):ti,ab,kw OR ("triglycerides glucose (TyG) index"):ti,ab,kw OR ("triglycerides-glucose index"):ti,ab,kw OR ("TyG index"):ti,ab,kw OR ("triglyceride-glucose index"):ti,ab,kw)

# Supplementary Tables and Figures

## Supplementary Tables

Table S1. The association between TyG index and hypertension in the cross-sectional study.

| **Hypertension** | **Cases/participants** | | **Model 1** | | **Model 2** | | **Model 3** | | **Model 4** | |
| --- | --- | --- | --- | --- | --- | --- | --- | --- | --- | --- |
|  |  |  | **OR (95%CI)** | **P value** | **OR (95%CI)** | **P value** | **OR (95%CI)** | **P value** | **OR (95%CI)** | **P value** |
| **^3^Re-defining hypertension as SBP ≥ 130mmHg or DBP ≥ 80mmHg** | | | | | | | | | | |
| TyG index | T1 (ref) | 507/1044 |  |  |  |  |  |  |  |  |
|  | T2 | 643/1045 | 1.694 (1.424-2.016) | <0.001 | 1.509 (1.259-1.808) | <0.001 | 1.512 (1.262-1.813) | <0.001 | 1.314 (1.088-1.586) | 0.004 |
|  | T3 | 762/1044 | 1.692 (1.544-1.854) | <0.001 | 1.578 (1.436-1.733) | <0.001 | 1.579 (1.436-1.735) | <0.001 | 1.423 (1.276-1.586) | <0.001 |
|  | T4 | 822/1044 | 1.577 (1.479-1.681) | <0.001 | 1.461 (1.367-1.562) | <0.001 | 1.460 (1.365-1.562) | <0.001 | 1.305 (1.198-1.422) | <0.001 |
| **^4^Additional adjustments for anti-hypertensive medications** | | | | | | | | | | |
| Hypertension | T1 (ref) | 344/1044 |  |  |  |  |  |  |  |  |
|  | T2 | 477/1045 | 1.709 (1.431-2.041) | <0.001 | 1.512 (1.257-1.820) | <0.001 | 1.513 (1.257-1.822) | <0.001 | 1.268 (1.005-1.598) | 0.045 |
|  | T3 | 583/1044 | 1.604 (1.468-1.753) | <0.001 | 1.499 (1.367-1.644) | <0.001 | 1.508 (1.375-1.655) | <0.001 | 1.261 (1.109-1.433) | <0.001 |
|  | T4 | 677/1044 | 1.554 (1.463-1.651) | <0.001 | 1.465 (1.375-1.561) | <0.001 | 1.481 (1.389-1.579) | <0.001 | 1.249 (1.130-1.381) | <0.001 |
| ISH | T1 (ref) | 135/1044 |  |  |  |  |  |  |  |  |
|  | T2 | 226/1045 | 1.858 (1.472-2.346) | <0.001 | 1.609 (1.263-2.051) | <0.001 | 1.596 (1.251-2.036) | <0.001 | 1.419 (1.095-1.839) | 0.008 |
|  | T3 | 270/1044 | 1.533 (1.368-1.717) | <0.001 | 1.409 (1.252-1.585) | <0.001 | 1.418 (1.259-1.596) | <0.001 | 1.259 (1.094-1.449) | 0.001 |
|  | T4 | 282/1044 | 1.356 (1.257-1.462) | <0.001 | 1.264 (1.167-1.368) | <0.001 | 1.266 (1.169-1.371) | <0.001 | 1.136 (1.019-1.266) | 0.021 |
| IDH | T1 (ref) | 25/1044 |  |  |  |  |  |  |  |  |
|  | T2 | 31/1045 | 1.246 (0.731-2.126) | 0.419 | 1.277 (0.743-2.196) | 0.377 | 1.274 (0.739-2.194) | 0.384 | 1.107 (0.626-1.959) | 0.727 |
|  | T3 | 42/1044 | 1.307 (1.017-1.681) | 0.037 | 1.384 (1.071-1.789) | 0.013 | 1.393 (1.078-1.801) | 0.011 | 1.225 (0.911-1.647) | 0.180 |
|  | T4 | 39/1044 | 1.165 (0.983-1.381) | 0.078 | 1.239 (1.036-1.483) | 0.019 | 1.241 (1.036-1.487) | 0.019 | 1.136 (0.898-1.437) | 0.289 |
| SDH | T1 (ref) | 90/1044 |  |  |  |  |  |  |  |  |
|  | T2 | 122/1045 | 1.401 (1.052-1.867) | 0.021 | 1.316 (0.983-1.761) | 0.065 | 1.318 (0.984-1.766) | 0.064 | 1.086 (0.797-1.481) | 0.601 |
|  | T3 | 166/1044 | 1.416 (1.235-1.622) | <0.001 | 1.374 (1.196-1.578) | <0.001 | 1.377 (1.198-1.582) | <0.001 | 1.195 (1.014-1.409) | 0.034 |
|  | T4 | 222/1044 | 1.420 (1.301-1.550) | <0.001 | 1.374 (1.255-1.503) | <0.001 | 1.383 (1.263-1.515) | <0.001 | 1.275 (1.126-1.444) | <0.001 |

^1^Abbreviation: ISH: isolated systolic hypertension; IDH: isolated diastolic hypertension; SDH: systolic-diastolic hypertension; OR: odds ratio; 95% CI: 95% confidence interval. ^2^ P-values were determined using the logistic regression. ^3^Hypertension was defined as SBP ≥ 130mmHg or DBP ≥ 80mmHg. Model 1: Unadjusted. Model 2: Adjusted for age, gender and education levels. Model 3: Model 2 plus adjustment for lifestyle factors (current smoking, current drinking, physical activity, and sedentary behavior). Model 4: Model 3 plus adjustment for concomitant diseases (overweight/obesity, diabetes, dyslipidemia and depressive symptoms). ^4^Hypertension was defined as SBP ≥ 140mmHg or DBP ≥ 90mmHg or on antihypertensive medication. ISH was defined as SBP ≥ 140mmHg and DBP < 90mmHg. IDH was defined as SBP < 140mmHg and DBP ≥ 90mmHg. SDH was defined as SBP ≥ 140mmHg and DBP ≥ 90mmHg. Model 1: Unadjusted. Model 2: Adjusted for age, gender and education levels. Model 3: Model 2 plus adjustment for lifestyle factors (current smoking, current drinking, physical activity, and sedentary behavior). Model 4: Model 3 plus adjustment for concomitant diseases (overweight/obesity, diabetes, dyslipidemia and depressive symptoms) and anti-hypertensive medications.

Table S2. Characteristics of studies included in the meta-analysis.


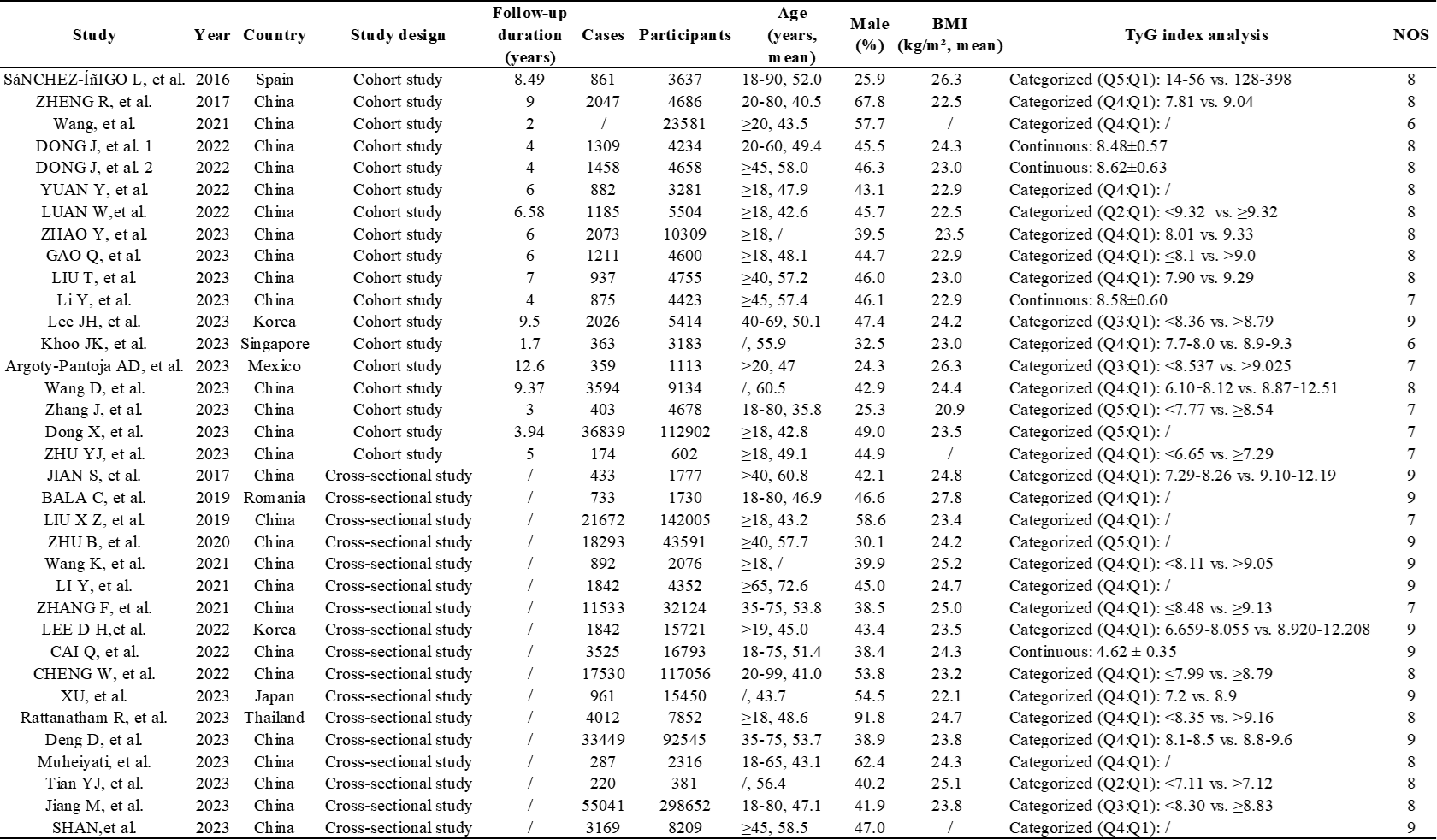


Table S3. Subgroup analysis of the association between TyG index and hypertension in the meta-analysis of cohort studies.

| **Factors stratified** | **N** | **HR (95% CI)** | **Heterogeneity** | | **P^2^** |  |
| --- | --- | --- | --- | --- | --- | --- |
|  |  |  | **I^2^ (%)** | **P^1^** |  |  |
| Overall analysis | 18 | 1.57 (1.25-1.96) | 97.10 | <0.001 |  |  |
| Age (years) |  |  |  |  | 0.109 |  |
| <45 | 5 | 1.81 (1.09-3.00) | 97.60 | <0.001 |  |  |
| ≥45 | 13 | 1.43 (1.30-1.58) | 72.20 | <0.001 |  |  |
| Gender |  |  |  |  | 0.406 |  |
| Male | 12 | 1.44 (1.07-1.93) | 97.60 | <0.001 |  |  |
| Female | 12 | 1.58 (1.16-2.14) | 97.20 | <0.001 |  |  |
| BMI (kg/m²) |  |  |  |  | 0.542 |  |
| <24 | 12 | 1.61 (1.16-2.23) | 97.80 | <0.001 |  |  |
| ≥24 | 5 | 1.41 (1.31-1.53) | 16.30 | 0.311 |  |  |
| Region |  |  |  |  | 0.680 |  |
| China | 14 | 1.54 (1.18-2.00) | 97.70 | <0.001 |  |  |
| Other | 4 | 1.63 (1.26-2.13) | 75.70 | <0.001 |  |  |
| ^1^P for heterogeneity within each subgroup with Q test. ^2^P for difference between subgroups with meta-regression analysis. | | | | | |  |
|  |  |  |  |  |  |  |

Table S4. Subgroup analysis of the association between TyG index and hypertension in the meta-analysis of cross-sectional studies.

| **Factors stratified** | **N** | **OR (95% CI)** | **Heterogeneity** | | **P^2^** |  |
| --- | --- | --- | --- | --- | --- | --- |
|  |  |  | **I^2^ (%)** | **P^1^** |  |  |
| Overall analysis | 18 | 2.01 (1.47-2.76) | 99.40 | <0.001 |  |  |
| Age (years) |  |  |  |  | 0.058 |  |
| <45 | 4 | 1.39 (1.01-1.92) | 93.80 | <0.001 |  |  |
| ≥45 | 14 | 2.21 (1.61-3.02) | 99.20 | <0.001 |  |  |
| Gender |  |  |  |  | 0.493 |  |
| Male | 8 | 1.73 (1.26-2.38) | 94.80 | <0.001 |  |  |
| Female | 8 | 2.06 (1.47-2.89) | 95.00 | <0.001 |  |  |
| BMI (kg/m²) |  |  |  |  | 0.707 |  |
| <24 | 7 | 1.91 (1.09-3.34) | 99.70 | <0.001 |  |  |
| ≥24 | 11 | 2.07 (1.67-2.56) | 93.20 | <0.001 |  |  |
| Region |  |  |  |  | 0.316 |  |
| China | 14 | 1.90 (1.31-2.77) | 99.50 | <0.001 |  |  |
| Other | 4 | 2.44 (1.70-3.49) | 93.30 | <0.001 |  |  |
| ^1^P for heterogeneity within each subgroup with Q test. ^2^P for difference between subgroups with meta-regression analysis. | | | | | |  |
|  |  |  |  |  |  |  |

## Supplementary Tables


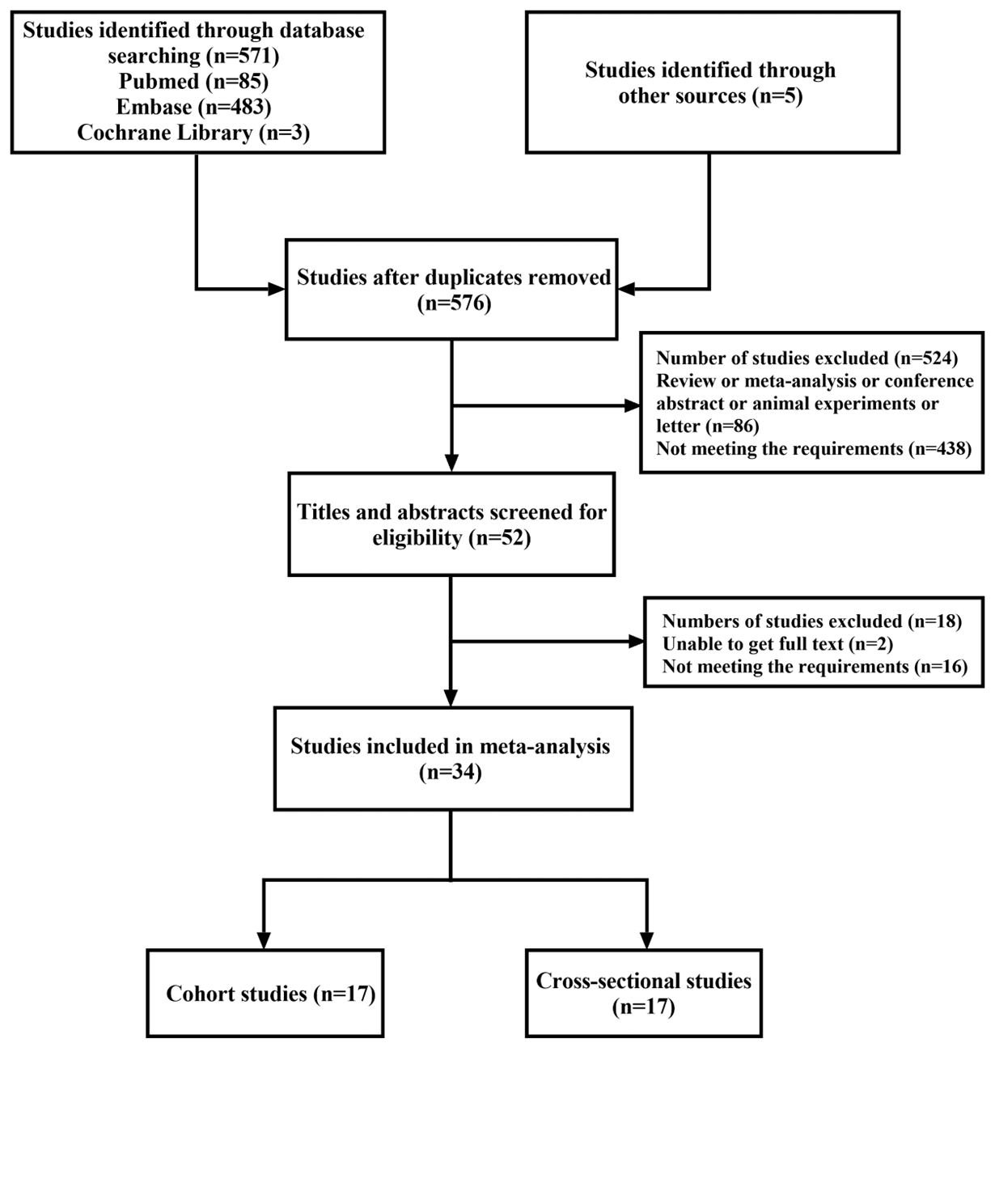


Figure S1. Flowchart of database search and selection.

Figure S2. Sensitivity analysis of the association between TyG index and hypertension in the meta-analysis of cohort studies.

Figure S3. Sensitivity analysis of the association between TyG index and hypertension in the meta-analysis of cross-sectional studies.


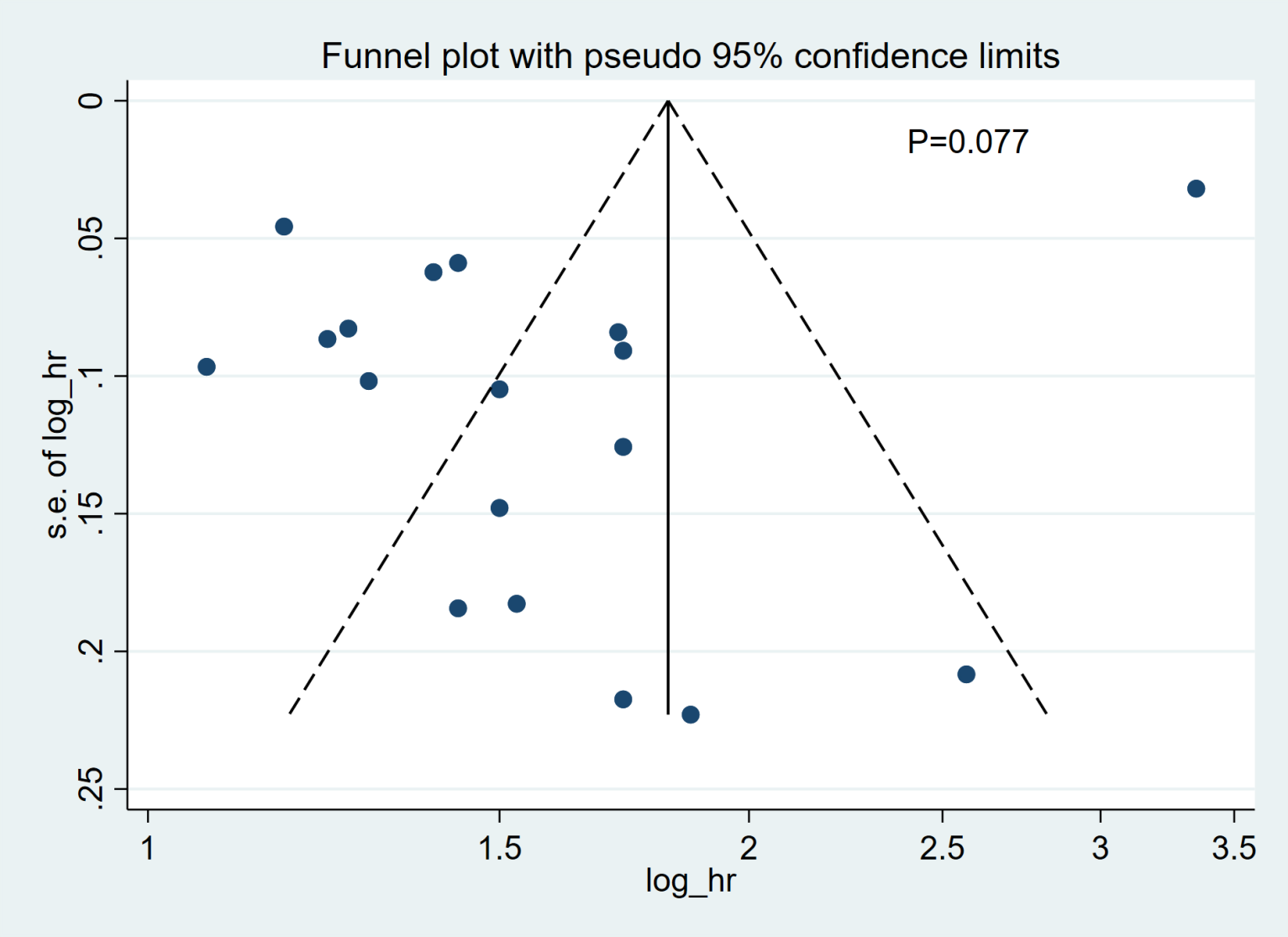


Figure S4. Funnel plot of the association between TyG index and hypertension in the meta-analysis of cohort studies.


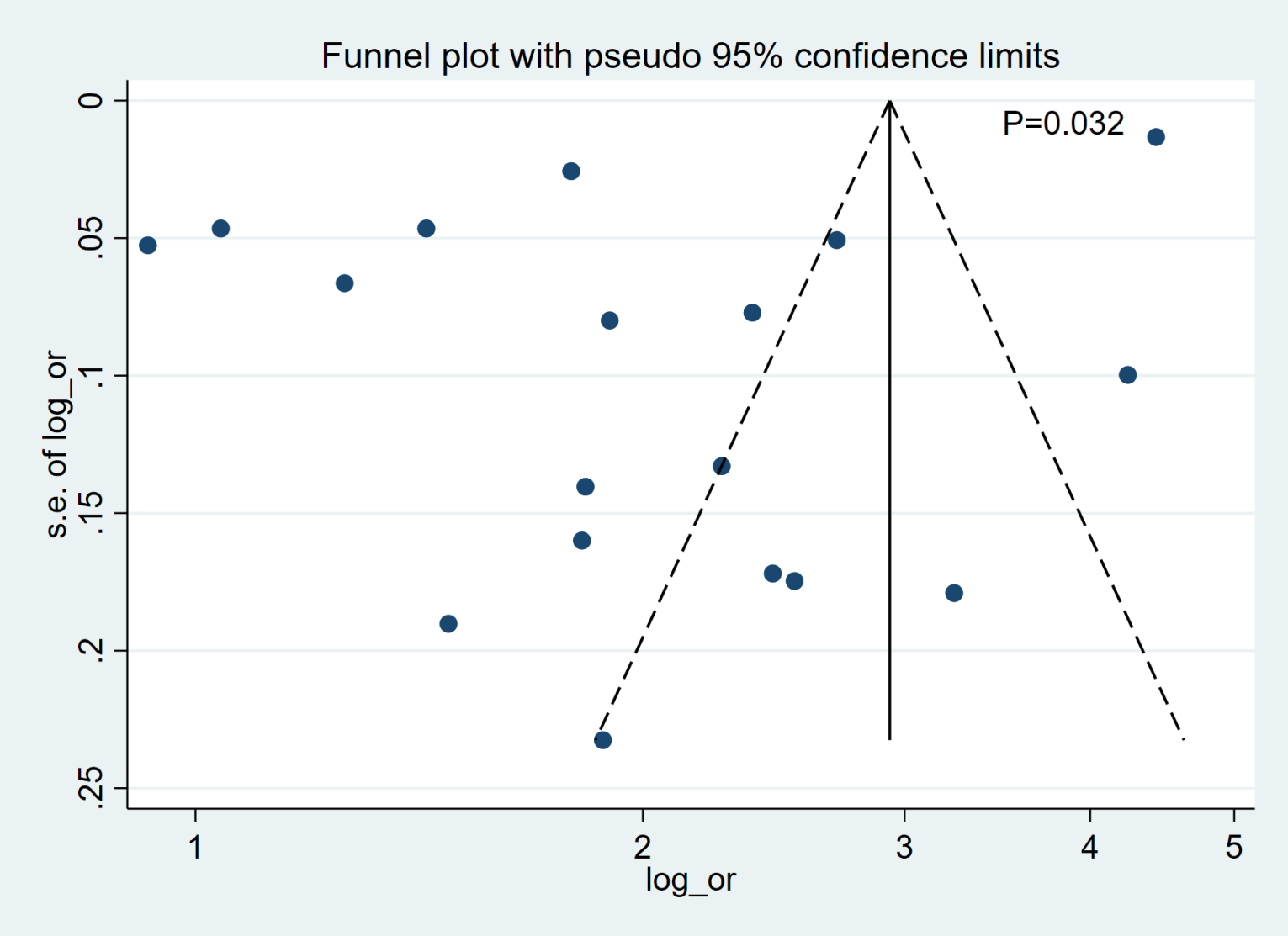


Figure S5. Funnel plot of the association between TyG index and hypertension in the meta-analysis of cross-sectional studies.

Figure S6. Egger’s funnel plot of the association between TyG index and hypertension in the meta-analysis of cohort studies.

Figure S7. Egger’s funnel plot of the association between TyG index and hypertension in the meta-analysis of cross-sectional studies.

Figure S8. Begg’s funnel plot of the association between TyG index and hypertension in the meta-analysis of cohort studies.

Figure S9. Begg’s funnel plot of the association between TyG index and hypertension in the meta-analysis of cross-sectional studies.
